# Modeling and Optimization of a Novel ScAlN-Based MEMS Scanning Mirror with Large Static and Dynamic Two-Axis Tilting Angles

**DOI:** 10.3390/s21165513

**Published:** 2021-08-17

**Authors:** Changhe Sun, Yufei Liu, Bolun Li, Wenqu Su, Mingzhang Luo, Guofeng Du, Yaming Wu

**Affiliations:** 1 School of Electronics and Information, Yangtze University, Jingzhou 434023, China; 202072656@yangtzeu.edu.cn (B.L.); 201801203@yangtzeu.edu.cn (W.S.); lmz@yangtzeu.edu.cn (M.L.); gfdu@yangtzeu.edu.cn (G.D.); 2 Cooperative Innovation Center of Unconventional Oil and Gas, Yangtze University, Ministry of Education, Wuhan 430100, China; 3 Key Laboratory of Optoelectronic Technology & Systems, Chongqing University, Ministry of Education, Chongqing 400044, China; Yufei.Liu@cqu.edu.cn; 4 State Key Laboratory of Transducer Technology, Shanghai Institute of Microsystem and Information Technology, Chinese Academy of Sciences, Shanghai 200050, China; yamingwu@mail.sim.ac.cn

**Keywords:** MEMS scanning mirror, ScAlN, piezoelectric actuator, rotation transformation, static tilting angle

## Abstract

The piezoelectric MEMS (micro-electro-mechanical systems) scanning mirrors are in a great demand for numerous optoelectronic applications. However, the existing actuation strategies are severely limited for poor compatibility with CMOS process, non-linear control, insufficient mirror size and small angular travel. In this paper, a novel, particularly efficient ScAlN-based piezoelectric MEMS mirror with a pupil size of 10 mm is presented. The MEMS mirror consists of a reflection mirror plate, four meandering springs with mechanical rotation transformation, and eight right-angle trapezoidal actuators designed in Union Jack-shaped form. Theoretical modeling, simulations and comparative analysis have been investigated for optimizing two different device designs. For Device A with a 1 mm-length square mirror, the orthogonal and diagonal static tilting angles are ±36.2°@200 V_DC_ and ±36.2°@180 V_DC_, respectively, and the dynamic tilting angles increases linearly with the driving voltage. Device B with a 10 mm-length square mirror provides the accessible tilting angles of ±36.0°@200 V_DC_ and ±35.9°@180 V_DC_ for horizontal and diagonal actuations, respectively. In the dynamic actuation regime, the orthogonal and diagonal tilting angles at 10 Hz are ±8.1°/V_pp_ and ±8.9°/V_pp_, respectively. This work confirmed that the Union Jack-shaped arrangement of trapezoidal actuators is a promising option for designing powerful optical devices.

## 1. Introduction

Micro-electro-mechanical systems (MEMS)-based scanning mirrors have been proven as an indispensable technology in laser radar [1], optical communication [2], optical switches [3], high-resolution displays [4] and biomedical imaging [5]. The main advantages of MEMS scanning mirrors over the conventional large-size mechanisms include: miniaturized structure, low-power consumption, easy integration and cost efficiency. Typically, a reliable MEMS scanning mirror, for instance used in light detection and ranging (LIDAR) imaging [1], is desired to provide a specified tilting angle and retain its position at arbitrary moment according to the applied voltage. Moreover, the ideal scanning mirror should have good mechanical linearity (>99%), high-accuracy control in the range above ±15° and a pupil size of 1 mm to 10 mm to achieve high resolution [1,6,7,8,9,10,11]. Until now, there have been a variety of actuation methods for MEMS scanning mirrors, mostly based on electrostatic, electromagnetic, electrothermal and piezoelectric actuation principles [12,13,14,15,16].

Owing to simple implementation, easy process integration and low power consumption, the electrostatic actuation has become a relatively mature method. The electrostatic actuator for MEMS scanning mirrors is essentially a capacitor with two parallel plates to produce the electrostatic force and then drive the mirror plate oscillation. Considering the fact that the parallel plate actuation force is proportional to the square of the voltage, a high voltage of several hundred volts is typically applied to target a large effective deflection and tilting angle. However, the pull-in phenomenon, which always occurs in the central region of the parallel plates under high voltage excitation, not only requires a strict compromise between the effective plate deflection and the driving voltage, but it also brings micromirror failure risk. Meanwhile, the electrostatic force with a non-linear relationship to its applied voltage undoubtedly makes the micromirror control complicated. To address this issue, some vertical electrostatic comb-drive actuators have been designed [10,17,18], while the complexities of the device structure and microfabrication process limit their practical applications. As one of the alternatives, the electromagnetic actuation based on the Lorentz force is also frequently adopted due to the large deflection at a low driving voltage and a high scanning frequency up to 15 kHz. The defects of this mechanism include high power consumption, complex microfabrication process and unidirectional actuation with regard to the soft magnetic micromirrors [1,14,19]. Li et al. developed a polymer composite-based hard-magnetic micromirror with bidirectional rotation, but the deflection angle of the micromirror ranges only between ±1.3° [20]. The electrothermal actuation is another important method for actuating MEMS scanning mirrors, which possesses good linear control performance and large deflection angle. Nevertheless, its intrinsic thermal dissipation, slow response, long-term instability and relatively high power consumption has made this option less attractive for implementation in portable and large array applications [19].

Although each actuation method above can exhibit some unique characteristics for certain applications, some common challenges confronted largely prevent the achievement of both large 2-degrees-of-freedom (2-DoF) tilting angles and static non-resonant operation. In comparison, numerous impressive piezoelectric actuation micromirrors reported in the previous literature have demonstrated the achievement of resonant/static actuation mode, linear control, fast response and low power consumption characteristics [4,11,16,21,22,23,24]. A piezoelectric micromirror is less affected by air damping, thermal or electromagnetic interference than those driven by the electrostatic, thermal or electromagnetic force. Besides the advantages in the actuation mechanisms, a wide variety of designs for piezoelectric MEMS mirrors have also been developed, with most of them deploying a large mechanical rotation angle and a low operation voltage level [4,11,16,22,23,24]. Such superior architectures are almost entirely based on bulk or thin PbZr_x_Ti_1−x_O_3_ (PZT) piezoelectric material to meet the requirements in laser projection and detection applications. For instance, Baran et al. developed a 1.4 mm-wide PZT thin-film actuation scanning mirror with the combination of mechanical amplification, exhibiting an optical scan angle of 38.5° at 24 V with the resonant frequency of 40 kHz [4]. Chen et al. presents a MEMS thin-PZT cantilever driven micro-lens actuator capable of delivering a large out-of-plane displacement of 145 μm at 22 V driving voltage, with a resonant frequency of 2 kHz [16]. Although commonly used PZT-type micromirrors exhibit large deflection due to high piezoelectric coefficients, the high permittivity resulting in low energy conversion efficiency, large temperature coefficient, low Curie point, hysteresis behavior, challengeable patterning process and poor compatibility with the mainstream complementary metal-oxide-semiconductor (CMOS) and/or MEMS process greatly limits its wide use in the MEMS area [4,20,21,22,23,24].

The AlN-based piezoelectric actuation strategy for a micromirror application has been presented and fabricated firstly by Shao et al., with a modified silicon-on-insulator-based MEMS process, demonstrating full CMOS compatibility, excellent linear control and various mirror movement modes [21]. However, both dynamic and static mirror operation modes of this device have the tilting angles of only about 0.2°/V and 0.005°/V, respectively, which is attributed to low piezoelectric coefficients of AlN material. Besides, a similar AlN-based micromirror with a larger aperture has also been developed [25], while its static analysis shows a very low mechanical rotation efficiency owing to the under-optimized actuator design. Based on Table 1, ScAlN, as a significantly higher performance piezoelectric material than AlN and PZT, can be used to deliver a large mechanical tilting angle of ±14° at 150 V_DC_ [11,26,27], in addition to the aforementioned inherent superiorities in AlN-based micromirrors. Although only a handful of ScAlN-based micromirrors have been created with the improvement of piezoelectric coefficient $e_{31,f}$ [11,28,29,30], almost all of them have a limited mirror plate with the aperture size of less than 1 mm. Generally, the large aperture size and mass of a MEMS scanning mirror limits the possibility of achieving a larger scanning angle and a higher operation frequency unless the actuation moment is high enough [9]. Up to now, to overcome the aforementioned limitations of PZT-, AlN- and ScAlN-based micromirrors, a MEMS scanning mirror with two-dimensional static and/or dynamic tilting angles of greater than ±15° in the centimeter range is desired and being pursued for laser projection applications [11,31]. In addition to improving the fabricate uniformity with low residual stress, raising the piezoelectric coefficient of ScAlN material and deploying multiple ScAlN layers, one of the most pressing areas of research needed to significantly promote MEMS mirror technology lies primarily in pushing novel advanced designs.

Aiming at further improving the theoretical scanning angle of the ScAlN-based MEMS mirror, a novel actuation mechanism with large two-axis tilting angles at both static and dynamic operation modes is developed in this paper. The structural description and operation principle of the actuator will be detailed in Section 2. The analytical modeling and analysis is presented in Section 3. The three-dimensional (3D) finite-element modeling (FEM) simulation and optimization is investigated and discussed in Section 4, followed by conclusions in Section 5.

## 2. Electromechanical Design of ScAlN-Based Piezoelectric Micro-Electro-Mechanical Systems (MEMS) Mirror

### 2.1. MEMS Mirror Structure

**Figure 1:**
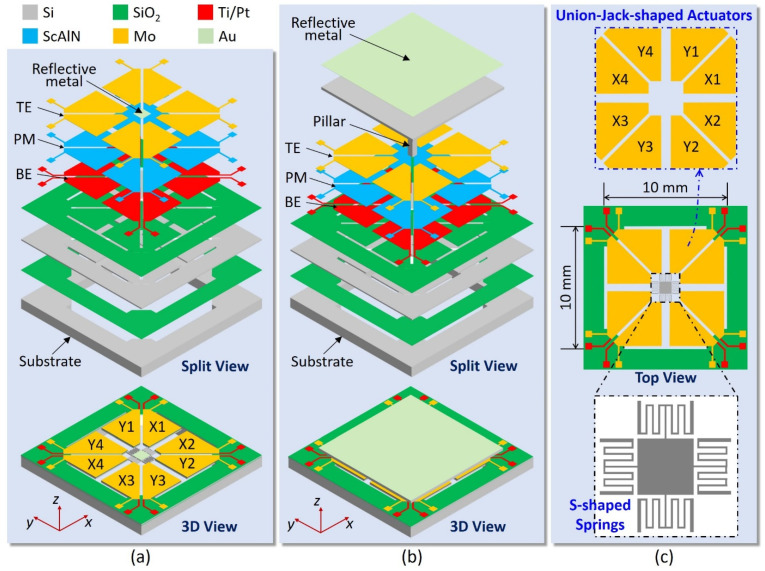
Architectures of two Sc_x_Al_1−x_N-based micro-electro-mechanical systems (MEMS) mirrors: (**a**) Device A, (**b**) Device B, (**c**) Union Jack-shaped actuators and S-shaped meandering springs.

Due to the aforementioned factors, a particularly efficient piezoelectric MEMS scanning mirror has been designed for investigating both the static and dynamic electromechanical performance using Sc_x_Al_1−x_N material as the actuator with Sc content up to x = 0.50. As illustrated in Figure 1, the architecture of the MEMS mirror (Device A) is composed of a square reflection mirror plate and eight trapezoidal Sc_x_Al_1−x_N-based piezoelectric microcantilevers (PMs) sandwiched between a 200 nm top electrode (TE) and a 200 nm bottom electrode (BE), which are connected to a silicon-on-insulator (SOI) substrate having a 10-μm-thick device layer. Both the insulating SiO_2_ layer and Sc_x_Al_1−x_N film have the thickness of 1 μm. The mirror plate is coated with 150 nm Au thin film to increase the reflective coefficient of the reflector. To explore a much larger mechanical tilting angle than ±15° within 150 V DC voltage, eight PMs are arranged as Union Jack-shaped actuators and four S-shaped meandering springs are adopted to connect the actuators and the mirror plate (referring to Figure 1c). Such spring design can not only avoid high stress concentrations in the corners, but also leverage its rotation transformation to enlarge the tilting angle of the MEMS mirror. Benefiting from this MEMS mirror design, good mode separation without crosstalk is more easily achieved when operating at dynamic resonance mode, especially at multiple resonance modes.

To further satisfy the light manipulation requirements from laser radar and vision science applications for an effective mirror region of roughly 10 × 10 mm^2^, a modified MEMS mirror design concept (Device B) based on Device A is presented in Figure 1b, where a square pillar is placed on the central holding platform driven by eight PM actuators to mechanically support the square mirror plate with a length of 10 mm and a thickness of 100 μm. The height of the pillar is determined by the maximum tilting angle, i.e., it must be set at above 2.4 mm to target 25° tilting angle. Note that the maximum principal stress within both devices A and B need be controlled at the level of 800 MPa to ensure no mechanical failure [4,11,33]. The geometrical parameter specifications of the proposed MEMS mirror devices A and B have been listed in Table 2.

### 2.2. Actuation Principle

**Figure 2:**
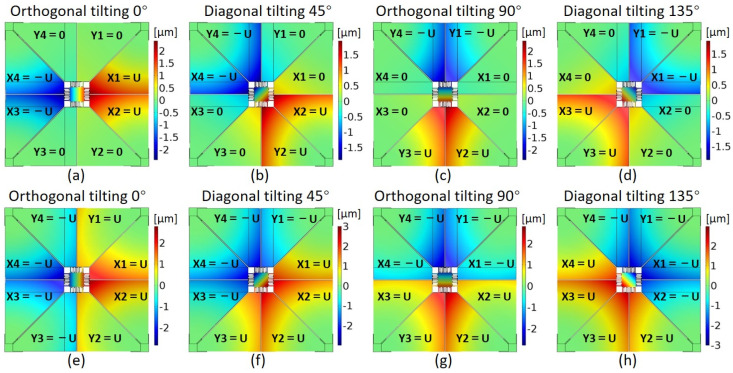
Mirror actuation principle for (**a**–**d**) 0°, 45°, 90° and 135°tilting angles when only using four PM actuators; (**e**–**h**) 0°, 45°, 90° and 135° tilting angles when using eight PM actuators.

When the driving voltages of PM actuators are distributed in an axisymmetric form, the induced actuation force applied on the proposed MEMS mirror will appear in pairs with opposite directions, resulting in antisymmetric deflections on two opposite sides of the MEMS mirror and a tilting rotation motion along its symmetrical axis. The main difference between these new mirror designs (A, B) and previously developed structures lies in the actuation principle: eight trapezoidal Sc_x_Al_1−x_N based PM actuators are fully deployed as bendable microcantilevers to efficiently deliver the bending forces and moments for arbitrary two-axis rotation motions of the MEMS mirror. The separate actuators design has also beneficial influence on the structure stiffness to adjust the dynamic resonance frequency of the mirror. The other advantage of such trapezoidal PM actuator design is to enlarge the effective actuation length of the microcantilever within the same device frame by fixing the actuator corners instead of their straight edges parallel to *x* or *y* axes. Based on COMSOL simulations, Figure 2 shows the mirror actuation principle for some targeted tilting angles (0°, 45°, 90° and 135°) in the cases of using only four PM actuators and all eight PM actuators. Although both two actuation cases are able to achieve arbitrary tilting angles, the eight PM actuators arrangement turns out to be more efficient from the aspect of the deflection, which can improve the output performance by about 50%.

## 3. Modeling and Analysis

**Figure 3:**
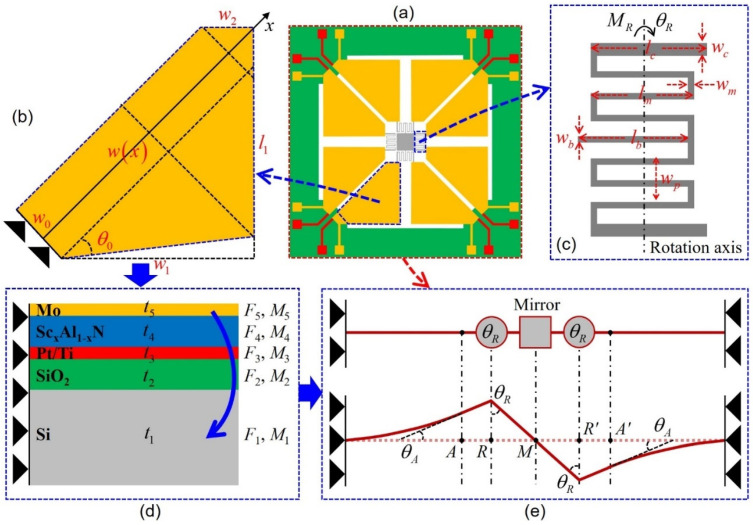
Simplified structural model of the trapezoidal Sc_x_Al_1−x_N based actuators: (**a**) structure configuration of the MEMS mirror, (**b**) top view of the PM actuator, (**c**) design schematic of the meandering spring, (**d**) cross-sectional view of the PM actuator, (**e**) simplification of the MEMS mirror.

**Figure 4:**
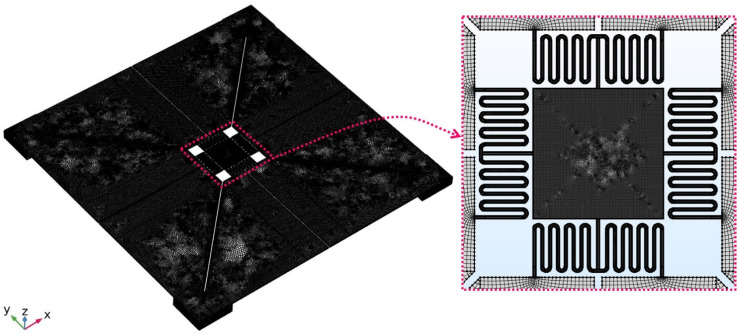
Meshing of the entire geometry of the proposed device.

For the designed MEMS mirrors, analytical modeling of the trapezoidal Sc_x_Al_1−x_N based actuators is essential to better understand their scanning characteristics and optimize the structural parameters. The static deflection and tilting angle at the bias voltage excitations are key performance indices to represent the device sensitivity. In the following mathematical models, each actuator is approximately modeled as a multilayer microcantilever with a fixed boundary condition at one end and a roller boundary condition at the meandering spring connection end, as shown in Figure 3, where the S-shaped meandering springs are functionalized as the rotation transformer. What needs illustration before modeling is that the right-angled trapezoidal actuators in Figure 4 are in a final designed form after optimization of structural angle *θ_0_* (i.e., *θ_0_* = 45°). In addition, to simplify the calculations, it is assumed that the initial residual stress caused during the microfabrication can be ignored and the radius of curvature resulting from the applied bending force is much larger than the total thickness of the multilayer trapezoidal actuator. Each layer of the actuator is in static equilibrium and the Sc_x_Al_1−x_N piezoelectric layer has already been polarized during theoretical analysis.

### 3.1. Analytical Model of the Static Behavior Multilayer Trapezoidal Actuator

The basic geometry of a multilayer trapezoidal actuator is illustrated in Figure 3. Such an actuator is comprised of one piezoelectric layer and four purely elastic layers, as a result, the conventional model of only single-layer piezoelectric actuator is not valid here.

According to the force and moment equilibrium conditions of the multilayer actuator, the total force $F_{total}$ and moment $M_{total}$ can be expressed as:

(1) $$F_{total}=\sum_{i=1}^{5}F_{i}=0$$

(2) $$M_{total}=\sum_{i=1}^{5}M_{i}+\sum_{i=1}^{5}F_{i}\left(\sum_{j=1}^{i-1}t_{j}+\frac{t_{i}}{2}\right)=0$$

where $t_{0}=0$, $t_{i}$ is the thickness of the *i*-th layer in the multilayer actuator, $F_{i}$ and $M_{i}$ are the force and moment at the cross section of the *i*-th layer shown in Figure 3d, respectively. According to the assumption that the actuator thickness is much less than the overall actuator curvature, the curvature *R* of each layer is approximately equal to each other, which can be given by [34]:

(3) $$\frac{1}{R}=\sum_{i=1}^{5}M_{i}/\sum_{i=1}^{5}C_{i}$$

(4) $$C_{i}=\frac{1}{3}E_{i}w\left(x\right)t_{i}\left[t_{i}^{2}+3\left(z_{n}-\sum_{j=1}^{i}t_{j}\right)\left(z_{n}-\sum_{j=1}^{i-1}t_{j}\right)\right]$$

(5) $$w\left(x\right)=\left\{ \begin{array}{l} w_{0}+x\tan\theta_{0},x\leq \sqrt{2}w_{1}/\left(1+\tan\theta_{0}\right)\\ w_{0}+\sqrt{2}w_{1}-x,x\leq w_{0}+\sqrt{2}\left(w_{1}-w_{2}\right)\\ 2w_{0}+\sqrt{2}\left(2w_{1}-w_{2}\right)-2x,x\leq L_{0} \end{array}\right.$$

where *E_i_* and *C_i_* represents the Young’s modulus and flexural rigidity of the *i*-th layer in the multilayer actuator, respectively, and $L_{0}=w_{0}+\sqrt{2}\left(w_{1}-w_{2}/2\right)$ is the effective actuation length of the multilayer actuator. The effective surface area of the actuator can be obtained by integrating its width *w*(*x*) along the length direction *x*, which is proportional to the structural angle *θ_0_* and reaches the maximum at *θ_0_* = 45°. As a result, the flexural rigidity *C_i_* of the *i*-th layer has a maximum value when *θ_0_* = 45°. *z_n_* is the neutral axis position, which is calculated according to:

(6) $$z_{n}=\frac{2\sum_{i=1}^{5}E_{i}t_{i}\sum_{j=1}^{i}t_{j}-\sum_{i=1}^{5}E_{i}t_{i}^{2}}{2\sum_{i=1}^{5}E_{i}t_{i}}$$

The bending moments produced in the multilayer actuator contains two part: one moment *M_4_* produced in the piezoelectric layer and one opposing moment *M_R_* associated to the torsional meandering spring. To model the deflection and bending angle of the piezoelectric actuator for the static operation case, we can firstly assume the bending angle at one spring connecting end of the piezoelectric layer is given as *θ_A_* and the angle at the other end of the torsional meandering spring is estimated as *θ_R_* under the torque *M_R_*. Therefore, the bending moment of the multilayer actuator is the difference between these two moments, given by:

(7) $$\sum_{i=1}^{5}M_{i}=M_{4}-M_{R}=E_{4}w\left(x\right)d_{31p}t_{4}V\left(z_{n}-\sum_{j=1}^{4}t_{j}+\frac{t_{4}}{2}\right)-M_{R}$$

(8) $$M_{R}=K_{\theta R}\left(\frac{\pi }{2}-\theta_{A}+\theta_{R}\right)$$

where *K_θR_* is the torsional spring constant, which is equal to the spring constant of all meandering spring segments in series.

Insertion of Equations (4) and (7) into (3) yields:

(9) $$\frac{1}{R}=\frac{3E_{4}d_{31p}t_{4}V\left(z_{n}-\sum_{j=1}^{4}t_{j}+\frac{t_{4}}{2}\right)}{\sum_{i=1}^{5}E_{i}t_{i}\left[t_{i}^{2}+3\left(z_{n}-\sum_{j=1}^{i}t_{j}\right)\left(z_{n}-\sum_{j=1}^{i-1}t_{j}\right)\right]}-\frac{K_{\theta R}\left(\frac{\pi }{2}-\theta_{A}+\theta_{R}\right)}{\sum_{i=1}^{5}C_{i}}$$

Equation (9) shows that the structural angle *θ_0_* should be optimized at 45° to obtain the best defection performance, which is highly consistent with the following simulated results. In order to derive *K_θR_* about the rotation axis with a flexural rigidity *C_R1_* of each horizontal spring segment and *C_R2_* of each vertical spring segment, it can be summarized as the following [16]:

(10) $$\frac{1}{K_{\theta R}}=8\frac{w_{p}-w_{m}}{C_{R1}}+\frac{6l_{m}+2l_{c}}{C_{R2}}$$

Combining Equations (9) and (10), the deflection ξ(x) of the actuator along the *x*-axis, in relation to the curvature *R*, is written as:

(11) $$\xi \left(x\right)=\frac{x^{2}}{2R},0\leq x\leq L_{0}$$

The bending angle *θ*(*x*) of the actuator can be yielded for small deflections as:

(12) $$\theta (x)=\frac{d\xi \left(x\right)}{dx}=\frac{x}{R},0\leq x\leq L_{0}$$

Considering that the bending angle *θ*(*x*) at the tip deflection of the actuator equals *θ_A_*, the rotation angle *θ_R_* of the meandering spring should meet the following relationship:

(13) $$\theta_{R}=\arcsin\left[\frac{\xi \left(L_{0}\right)+\left(l_{c}-l_{m}/2\right)\sin\theta_{A}}{l_{b}-l_{m}/2}\right]$$

Therefore, the statically driven mirror rotation angle *θ_R_* for arbitrary DC voltage excitation can be calculated and optimized by means of integration of Equations (9)–(12) according to (13), which is a function of structural parameters of the multilayer trapezoidal actuator.

### 3.2. Analytical Model of the Dynamic Behavior Multilayer Trapezoidal Actuator

In addition to the static behavior of the multilayer trapezoidal actuator, the analytical prediction of its dynamic behavior is also of significance. When the multilayer trapezoidal actuator operates at the bending vibration eigenmodes with the damping *r_a_*, the differential equation for bending motions of the actuator with the external load *f*(*x*,*t*) can be given by:

(14) $$C\frac{\partial^{4}\xi \left(x,t\right)}{\partial x^{4}}+\mu \frac{\partial^{2}\xi \left(x,t\right)}{\partial t^{2}}+r_{a}\frac{\partial \xi \left(x,t\right)}{\partial t}=f\left(x,t\right)$$

where $\xi \left(x,t\right)=\xi \left(x\right)\phi \left(t\right)=\sum_{m=1}^{\infty }\xi_{m}\left(x\right)\phi_{m}e^{j\omega_{m}t}$ which absolutely converges as *m* increases to ∞, $C=\sum_{i=1}^{5}C_{i}$ represents the total flexural rigidity over the length direction of the actuator, $\omega_{m}$ physically corresponds to the *m*-th natural bending vibration mode, μ is defined as follows:

(15) $$\mu =\sum_{i=1}^{5}\rho_{i}t_{i}w\left(x\right)$$

The general solution ξ(x) of Equation (14) results from the fixed-roller boundary conditions of the multilayer trapezoidal actuator, which are represented by:

(16) $$\xi \left(0\right)=0,\frac{d\xi \left(0\right)}{dx}=0$$

(17) $$\frac{d^{2}\xi \left(L_{0}\right)}{dx^{2}}=-\frac{M_{4}-M_{R}}{C},\frac{d^{3}\xi \left(L_{0}\right)}{dx^{3}}=-\frac{M_{R}}{C\left(l_{b}-l_{m}/2\right)}$$

The *m*-th natural vibration mode frequency of the actuator can be determined by:

(18) $$\omega_{m}=\frac{a_{m}^{2}}{L_{0}^{2}}\sqrt{\frac{C}{\mu }}$$

where $a_{m}$ denotes the characteristic zeros of the *m*-th natural vibration mode.

In combination with Equations (16) and (17), the deflection $\xi_{m}\left(x\right)$ of the *m*-th natural vibration mode can be determined by adopting the linearly independent Rayleigh functions for solving Equation (14) [35]:

(19) $$\xi_{m}\left(x\right)=\frac{1}{2}\left[\cosh\left(\frac{a_{m}x}{L_{0}}\right)-\cos\left(\frac{a_{m}x}{L_{0}}\right)\right]-\frac{1}{2}b_{m}\left[\sinh\left(\frac{a_{m}x}{L_{0}}\right)-\sin\left(\frac{a_{m}x}{L_{0}}\right)\right]$$

where $b_{m}$ is defined by:

(20) $$b_{m}=\frac{\cosh\left(a_{m}\right)+\cos\left(a_{m}\right)}{\sinh\left(a_{m}\right)+\sin\left(a_{m}\right)}$$

Taking the orthogonality properties for bending waves arising on the bending actuator into account, multiplication on both sides of Equation (14) with $\xi_{m}\left(x\right)$ and following integration with respect to the actuator length $L_{0}$ results in [10]:

(21) $${\ddot{\phi }}_{m}+2\varsigma_{m}\omega_{m}{\dot{\phi }}_{m}+\omega_{m}^{2}\phi_{m}=\frac{\int_{0}^{L_{0}}f\left(x,t\right)\xi_{m}\left(x\right)dx}{\mu \int_{0}^{L_{0}}\xi_{m}^{2}\left(x\right)dx}$$

where $r_{a}/\mu =2\varsigma_{m}\omega_{m}$, $\varsigma_{m}$ denotes a dimensionless attenuation constant. Equation (21) determines the temporal characteristics of the bending vibration eigenmode $\xi_{m}\left(x\right)$ taking the damping $r_{a}$ into consideration.

The mechanical stresses originate from the piezoelectric layer and are referred to as piezoelectric moment *M_4_* in accordance with Equation (7). The harmonic driving voltage *V*(*t*) is applied over the whole piezoelectric bending actuator, thus the resulting load *f*(*x*,*t*) can be defined by the Dirac delta function, written as:

(22) $$f\left(x,t\right)=M_{4}\cos\left(\Omega t\right)\left[\frac{d\delta }{dx}\left(x-L_{0}\right)-\frac{d\delta }{dx}\left(x\right)\right]$$

In combination with the fixed-roller boundary conditions and Equation (22), Equation (21) results in:

(23) $${\ddot{\phi }}_{m}+2\varsigma_{m}\omega_{m}{\dot{\phi }}_{m}+\omega_{m}^{2}\phi_{m}=\frac{-M_{4}\cos\left(\Omega t\right)\xi^{\prime }(L_{0})}{\mu \int_{0}^{L_{0}}\xi_{m}^{2}\left(x\right)dx}$$

Since the attenuation constant $\varsigma_{m}$ is different from zero, the homogeneous solution decays in time. After expiration of the transient time, the oscillation $\phi_{m}\left(t\right)$ yields the particular solution:

(24) $$\phi_{m}\left(t\right)=\frac{-\xi^{\prime }(L_{0})}{\omega_{m}^{2}\mu \int_{0}^{L_{0}}\xi_{m}^{2}\left(x\right)dx\sqrt{{\left(1-\Omega^{2}/\omega_{m}^{2}\right)}^{2}+{\left(2\varsigma_{m}\Omega /\omega_{m}\right)}^{2}}}M_{4}\cos\left(\Omega t-\psi_{m}\right)$$

where the quantity denotes the phase angle in accordance with:

(25) $$\tan\psi_{m}=\frac{2\varsigma_{m}\Omega /\omega_{m}}{1-\Omega^{2}/\omega_{m}^{2}}$$

Thus, the spatial and temporal deflection of the piezoelectric bending actuator yields:

(26) $$\xi \left(x,t\right)=\sum_{m=1}^{\infty }\frac{-\xi \left(x\right)\xi^{\prime }(L_{0})}{\omega_{m}^{2}\mu \int_{0}^{L_{0}}\xi_{m}^{2}\left(x\right)dx\sqrt{{\left(1-\Omega^{2}/\omega_{m}^{2}\right)}^{2}+{\left(2\varsigma_{m}\Omega /\omega_{m}\right)}^{2}}}M_{4}\cos\left(\Omega t-\psi_{m}\right)$$

By means of differentiation of the deflection ξ(x,t) with respect to *x*, the bending angle $\theta_{A}\left(t\right)$ at the tip of the piezoelectric actuator results in:

(27) $$\theta_{A}\left(t\right)=\sum_{m=1}^{\infty }\frac{-{\left[\xi^{\prime }(L_{0})\right]}^{2}}{\omega_{m}^{2}\mu \int_{0}^{L_{0}}\xi_{m}^{2}\left(x\right)dx\sqrt{{\left(1-\Omega^{2}/\omega_{m}^{2}\right)}^{2}+{\left(2\varsigma_{m}\Omega /\omega_{m}\right)}^{2}}}M_{4}\cos\left(\Omega t-\psi_{m}\right)$$

In combination with Equation (13), the dynamically driven mirror rotation angle *θ_R_*(*t*) for a harmonic voltage excitation can be predicted.

## 4. Three-Dimensional (3D) Finite-Element Modeling (FEM) Simulation, Optimization and Discussion

To better understand the static and dynamic characteristics of the proposed devices A and B, 3D FEM analysis and optimization were performed using Multiphysics COMSOL commercial simulation software. The mechanical and piezoelectric properties of the Sc_x_Al_1−x_N layer with the Sc content *x* ranging from 0% to 50% can be approximately predicted by the following Equations (28) and (29) [27,36,37,38], while the material properties of the other layers used in the simulations are listed in Table 3. It is noted that both the mechanical damping and dielectric loss within the Sc_x_Al_1−x_N layer have been considered as 0.01 during simulations in view of actual conditions [11,26,37]. The meshing of the entire geometry has been taken by the sweep mesh with the quadrilateral source faces, as depicted in Figure 4. The number of domain elements is about 0.5 million with the maximum and average growth rates of roughly 3.4 and 1.1, respectively. The number of degrees of freedom solved for the model is about 9 million. Besides, the fixed boundaries at the lower surfaces of the silicon substrate are used for the FEM models. The relative tolerance during the simulation studies is set at 0.01 to balance the computation speed and accuracy.

(28) $$\begin{array}{l} C_{11}\left(x\right)=410.2\left(1-x\right)+295.3x-210.3x\left(1-x\right)\\ C_{12}\left(x\right)=142.4\left(1-x\right)+198.6x-61.9x\left(1-x\right)\\ C_{13}\left(x\right)=110.1\left(1-x\right)+135.5x+78.9x\left(1-x\right)\\ C_{33}\left(x\right)=385\left(1-x\right)-23.8x-101.4x\left(1-x\right)\\ C_{44}\left(x\right)=122.9\left(1-x\right)+169.5x-137.3x\left(1-x\right)\\ C_{66}\left(x\right)=0.5C_{11}\left(x\right)-0.5C_{12}\left(x\right) \end{array}$$

(29) $$\begin{array}{l} d_{15}\left(x\right)=-3.17+0.487x+0.660x^{2}-0.746x^{3}\\ d_{31}\left(x\right)=-2.14-15.09x+78.8x^{2}-229x^{3}\\ d_{33}\left(x\right)=5.02+42.3x-238x^{2}+704x^{3} \end{array}$$

### 4.1. Structure Optimization

**Figure 5:**
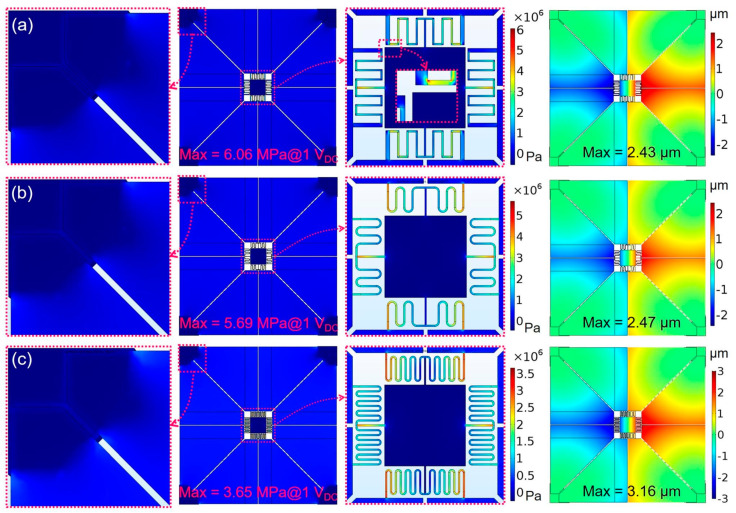
FEM simulations of material principal stress and defection in three piezoelectric MEMS mirrors with different meandering spring designs at 1 V_DC_ driving voltage: (**a**) rectangular-shaped spring, (**b**,**c**) S-shaped springs.

In order to achieve a static tilting angle of greater than ±15°, the piezoelectric MEMS mirror is typically required to be driven at a DC voltage up to 200 V, which will increase the risk of the device failure notably due to the maximum material stress beyond the break strength for the Si layer. The reachable tilting angles of the proposed Devices A and B are restricted by the stress limit of the materials. To avoid any breaking down behavior, maximum principal stress of less than 800 MPa is expected, in other words, the maximum principal stress per 1 V_DC_ voltage should be no higher than 4.0 MPa. Therefore, the first structural optimization point is the maximum principal stress within the piezoelectric MEMS mirror. Accordingly, FEM simulations at 1 V_DC_ voltage were performed for three different meandering spring designs, as shown in Figure 5. Note that the Sc content in Sc_x_Al_1−x_N film is assumed as 41% which has been processed and tested to provide a piezoelectric coefficient $e_{31,f}$ of about 2.83 C/m^2^ in several previous works of literature [27,32,37,38]. By comparing the results, it is obviously demonstrated that the S-shaped meandering springs in Figure 5b,c have less maximum principal stress than the rectangular-shaped meandering spring in Figure 5a. In addition, the maximum principal stress can be further reduced and the maximum mechanical deflection can be improved to some extent by properly decreasing the spring pitch and increasing the number of the spring turns. The S-shaped meandering spring design in Figure 5c has been adopted in all subsequent simulations.

**Figure 6:**
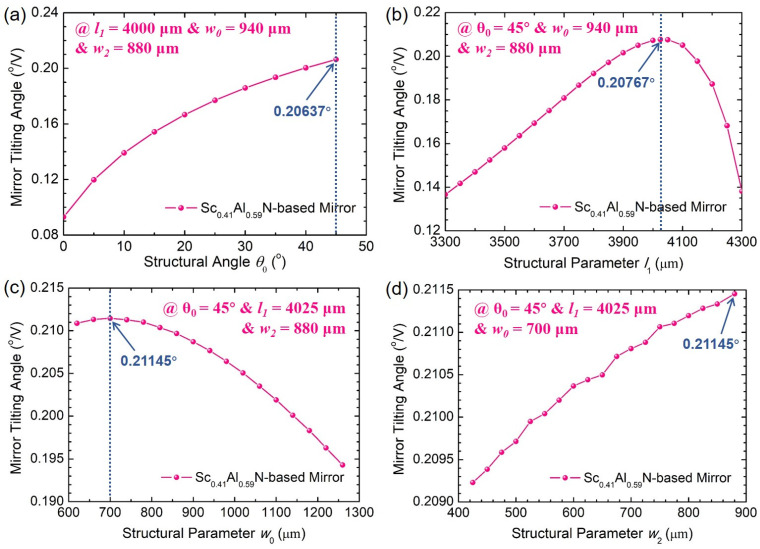
The dependence of the static tilting angles on the structural parameters: (**a**) *θ_0_*, (**b**) *l_1_*, (**c**) *w_0_* and (**d**) *w_2_*.

The second structural optimization point is for the piezoelectric MEMS actuator, which plays an important role in improving the reachable tilting angles. Based on the theoretical Equations (11)–(13), the mirror rotation angle *θ_R_* depends critically on the structural parameters *θ_0_*, *l_1_*, *w_0_* and *w_2_* which are independent and irrelevant to each other. Thus, these four structural parameters can be optimized individually by using a control variate method with three out of four structural parameters fixed for each parametric sweep. According to the simulation results shown in Figure 6, the structural angle *θ_0_* of the multilayer trapezoidal actuator is optimized at 45° when *l_1_* = 4000 μm, *w_0_* = 940 μm and *w_2_* = 880 μm, defining the actuator as right-angled trapezoidal shape, which is highly consistent with the above theoretical analysis. Moreover, there is a peak value for both the structural parameters *l_1_* and *w_0_*. After further optimizing structural parameter *w_2_* in its range, it can be seen from Figure 6d that the static tilting angle of the MEMS mirror reaches the threshold value of 0.21145°/V_DC_. The optimal values of structural parameters *l_1_*, *w_0_* and *w_2_* are given in Table 2.

**Figure 7:**
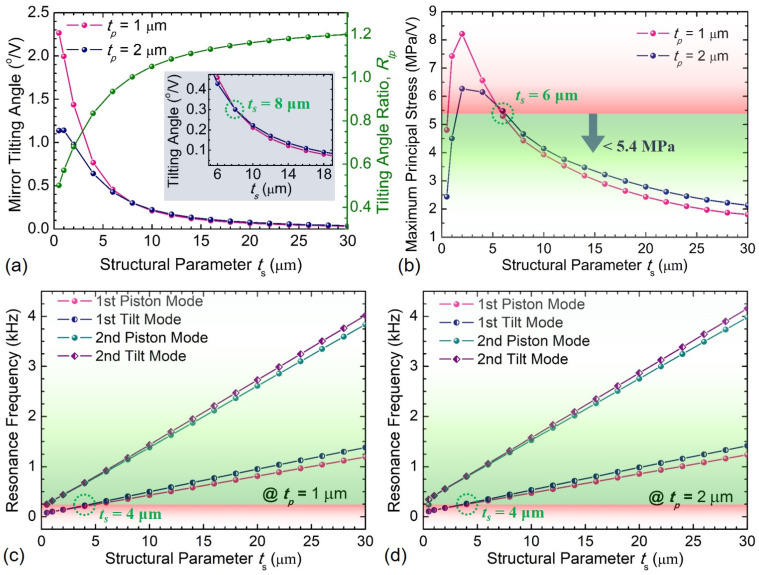
Simulation results of the MEMS scanning mirrors varying with the thickness *t_s_* of the Si device layer: (**a**) mirror tilting angle, (**b**) maximum principal stress, (**c**,**d**) resonance frequencies of different modes.

The thickness of the piezoelectric film and Si device layer is another important structural parameter to determine the mechanical tilting angle and frequency response. With increasing the thickness *t_s_* of the Si device layer, it is demonstrated from simulation results shown in Figure 7 that the MEMS scanning mirror will deliver a smaller mechanical tilting angle and linearly increase resonance frequencies for both piston modes and tilt modes. To obtain a more powerful Sc_x_Al_1−x_N-based scanning mirror from the perspective of structural design, deploying a thicker Sc_x_Al_1−x_N film to reduce electrical field intensity and induced stress may be an alternative approach [11]. The mechanical tilting angles and maximum principal stress of the MEMS scanning mirrors with 1 μm-thick and 2 μm-thick Sc_0.41_Al_0.59_N films are shown in Figure 7a,b. The tilting angle ratio *R_tp_* is defined as the ratio of the mirror tilting angles of two device designs with 1 μm-thick and 2 μm-thick Sc_0.41_Al_0.59_N films. The tilting angle ratio *R_tp_* increases with the thickness of the Si device layer and can be improved by about 20% when the thickness of the Si device layer is larger than 20 μm. The simulation results indicate that a 2 μm-thick Sc_0.41_Al_0.59_N based mirror can work more efficiently than a 1 μm-thick Sc_0.41_Al_0.59_N based mirror only when the thickness of the Si device layer is not less than 10 μm, because the thickness of Sc_0.41_Al_0.59_N film will make a significant impact on the total flexural rigidity of the multilayer actuator when the Si device layer is too thin, thereby affecting the mechanical deflection and tilting angle. Thus the thickness of the Sc_0.41_Al_0.59_N and Si device layers are set as 1 μm and 10 μm in all subsequent simulations, respectively.

**Figure 8:**
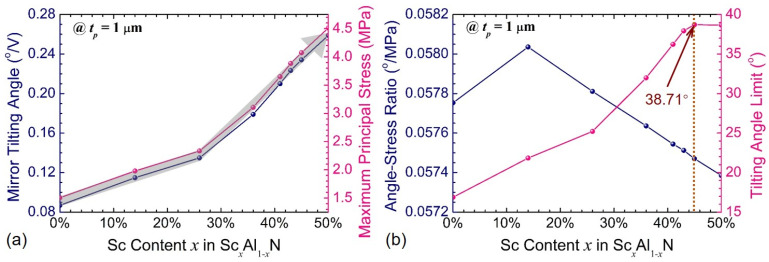
Dependence of the mechanical tilting angle and maximum principal stress on the Sc content: (**a**) mirror tilting angle, (**b**) angle-stress ratio.

In addition to the optimization of the structural parameters, the influence of the Sc content in Sc_x_Al_1−x_N film on the mechanical efficiency of the MEMS scanning mirror has also been considered. As shown in Figure 8a, both the mechanical tilting angle and maximum principal stress increases piecewise linearly with the Sc content changing from 0% to 50%. Considering the limitations that the maximum principal stress and driving DC voltage of the designed mirrors should be controlled within 800 MPa and 200 V_DC_ [16], respectively, it is necessary to strike a better balance between them in order to achieve its full potential. The ratio of the tilting angle and maximum principal stress for Sc_x_Al_1−x_N -based mirrors with different Sc contents is defined to demonstrate the sensitivity to the material stress, as shown in Figure 8b. By analyzing the attainable tilting angle subject to the above limitations, it is suggested that the best performance of the designed mirror may be realized when the Sc content in Sc_x_Al_1−x_N film increases to 45~50%. However, according to the previously reported experiments in terms of ScAlN crystal structure and piezoelectric response [36,37,38,41], both hexagonal wurtzite and cubic rocksalt phases coexist when the Sc content is between 42% and 45% and the crystal orientation drastically decreases when the Sc content is above 45%, implying that the optimal value of the Sc content in the proposed Sc_x_Al_1−x_N-based mirrors is within range of 41~45%. Moreover, the manufacturing process for achieving the scandium concentration of higher than 42% is really challenging in the present studies. Alternatively, the Sc content of 41% is considered in all subsequent simulations to further investigate both the static and dynamic performance of the designed mirrors.

### 4.2. Static Actuation

**Figure 9:**
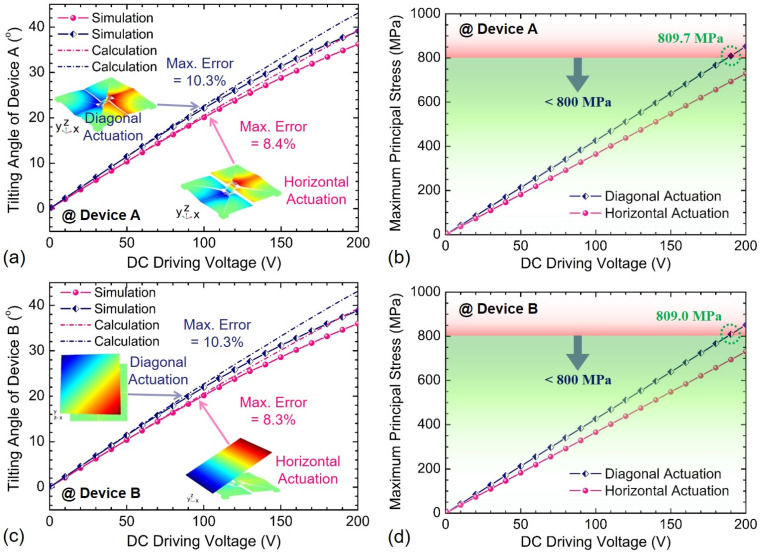
Comparison of dependence of the tilting angle and maximum principal stress on the DC driving voltages: (**a**,**b**) Device A without gravity, (**c**,**d**) Device B without gravity.

After above structure optimization for Devices A and B, the optimal design parameters are determined and listed in Table 2. The static performance of both devices are firstly simulated at the static actuation modes referring to the modal shapes given in Figure 2 regardless of gravity effect, as shown in Figure 9. Both the maximum principal stress and static tilting angles almost increase linearly with the DC driving voltage changing from 0 to 200 V, with linearity of greater than 0.995. For the horizontal actuation of Device A, the tilting angle reaches up to 36.2°@200 V_DC_ with a maximum principal stress of 729.8 MPa. As a comparison, the maximum tilting angle will be limited to 36.2°@180 V_DC_ within the maximum principal stress of 767.2 MPa for the diagonal actuation of Device A, because the maximum principal stress of 852.4 MPa at 200 V_DC_ will irreversibly destroy the device. The tilting angle sensitivities of Device A in horizontal and diagonal actuations are 0.1856°/V_DC_ and 0.2011°/V_DC_ in the DC voltage range from 0 to 180 V_DC_, respectively. Besides, the numerical calculations for both horizontal and diagonal tilting angles of Device A have also been made in the DC driving voltage range of 0 to 200 V, offering the theoretical tilting angle sensitivities of 0.1964°/V_DC_ and 0.2157°/V_DC_, respectively. The maximum relative errors between simulations and calculations are 8.4% for horizontal actuation and 10.3% for diagonal actuation at 200 V_DC_. The ratios of the diagonal to horizontal maximum principal stress and von Mises stress at the same DC voltage are about 117% and 140%, respectively, which may be caused by the result of two orthogonal stresses. On the other hand, the diagonal actuation can deliver a marginally higher static tilting angle sensitivity than the horizontal actuation owing to the softer torsion springs. Moreover, the static performance of Device B is also analyzed, showing very similar characteristics to Device A. The accessible tilting angles for both horizontal and diagonal actuations of Device B are 36.0°@200 V_DC_ and 35.9°@180 V_DC_, respectively. The tilting angle sensitivities of Device B in horizontal and diagonal actuations are 0.1843°/V_DC_ and 0.1999°/V_DC_ in the DC voltage range from 0 to 180 V_DC_, respectively, which are only slightly smaller than those of Device A. The explanation for the angle variation between two devices can be owing to the different mirror structures, albeit with the same piezoelectric actuators. Moreover, numerical calculations for both horizontal and diagonal tilting angles of Device B have also been undertaken in the DC driving voltage range of 0 to 200 V, offering the theoretical tilting angle sensitivities of 0.1949°/V_DC_ and 0.2141°/V_DC_, respectively. The maximum relative errors between simulations and calculations for Device B were 8.3% for horizontal actuation and 10.3% for diagonal actuation at 200 V_DC_.

**Figure 10:**
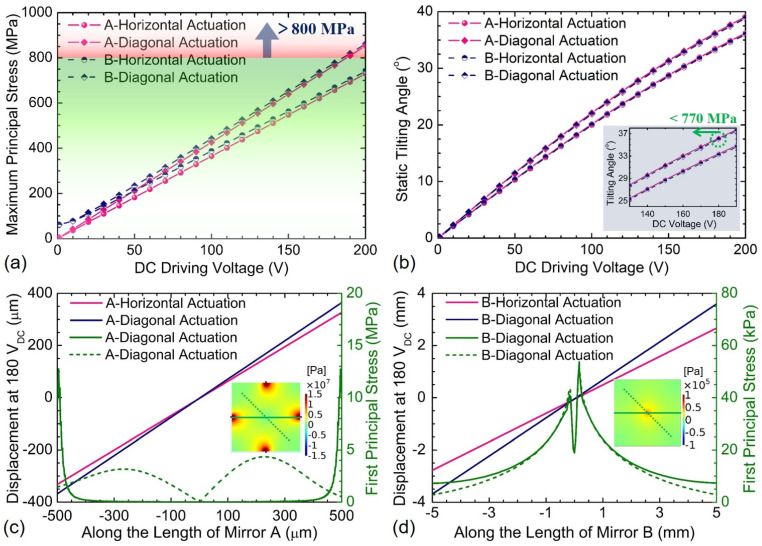
Comparison of dependence of the maximum principal stress and tilting angle on the DC driving voltages for Devices A and B with gravity: (**a**) maximum principal stress, (**b**) static tilting angle, (**c**,**d**) displacement.

Generally, the weight impact of the millimeter-scale mirror device on its static defection performance is too small to be considered. In order to illustrate such an argument, more detailed simulations were undertaken for validation. When taking into account the gravity effect, the dependence of the material stress and tilting angle on the DC driving voltages for Devices A and B are presented in Figure 10. It is observed that both devices have excellent linearity in maximum principal stress, though in comparison Device B suffers from about 10 MPa higher stress at the same driving voltage due to the larger mirror. The static tilting angle responses of the two devices also exhibit approximately linear increase tendency in the DC voltage range from 0 to 180 V_DC_. The tilting angle sensitivities of Device A in horizontal and diagonal actuations are 0.1856°/V_DC_ and 0.2010°/V_DC_, respectively, while those of Device B in horizontal and diagonal actuations are 0.1841°/V_DC_ and 0.1996°/V_DC_, respectively. After comparing two different cases with and without consideration of the device weight, the tilting angle sensitivity variations of both devices are only within 0.2%, which is consistent with the above argument. The displacement and inner principal stress distribution along the length of both mirrors (A, B) are plotted in Figure 10c,d. The maximum principal stresses within both mirrors occur in the four spring connection points and the central region, respectively, which are much lower than the residual stress of some fabricated micromirrors [15,16,38,40]. Moreover, the displacement curves are almost perfectly linear, indicating that there are no noticeable mechanical deformations occurring during the tilting motions of both mirrors (A, B). Therefore, the gravity effect of the mirrors during the analysis can be ignored to simplify theoretical calculations and simulations. Table 4 compares the static performance of Devices A and B presented in this paper with other piezoelectric scanning mirrors reported in the literature [11,21,42,43,44]. It is demonstrated that the proposed devices tend to exhibit an outstanding *θ*·*D* product.

### 4.3. Dynamic Actuation

**Figure 11:**
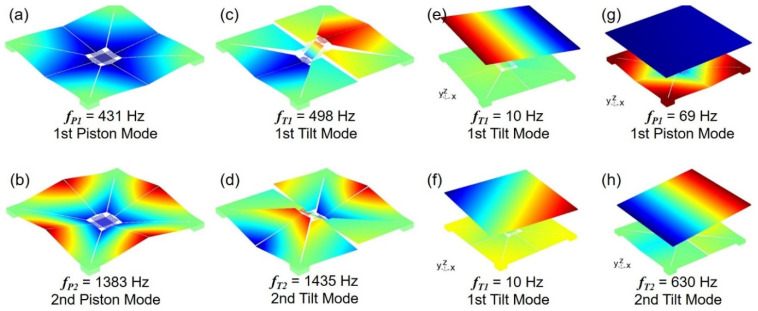
Simulated piston and tilt vibration modes: (**a**–**d**) Device A; (**e**–**h**) Device B.

Both the piston and tilt vibration modes are commonly used to steer or manipulate the reflection and phase of the light. To characterize the dynamic response of the proposed MEMS scanning mirrors (Devices A and B), the modal analysis was done firstly with 3D FEM simulations, as shown in Figure 11. This shows the first-order and second-order piston mode frequencies are 431 Hz and 1383 Hz for Device A, respectively, while the first-order and second-order tilt mode frequencies are 498 Hz and 1435 Hz, respectively. The modal frequencies of larger than 400 Hz for both piston and tilt vibration modes indicates the proposed Union Jack-shaped actuators have large enough stiffness to maintain regular operation. As for Device B, the frequencies of the first-order orthogonal and diagonal tilt vibration modes have dropped to 10 Hz, while the second-order tilt mode frequency can reach to 630 Hz. Moreover, the frequency of the first-order piston vibration mode is 69 Hz. As illustrated in the mirror actuation principle (Figure 2), these different vibration modes can be excited individually or in combination with proper arrangement of the PM actuators.

**Figure 12:**
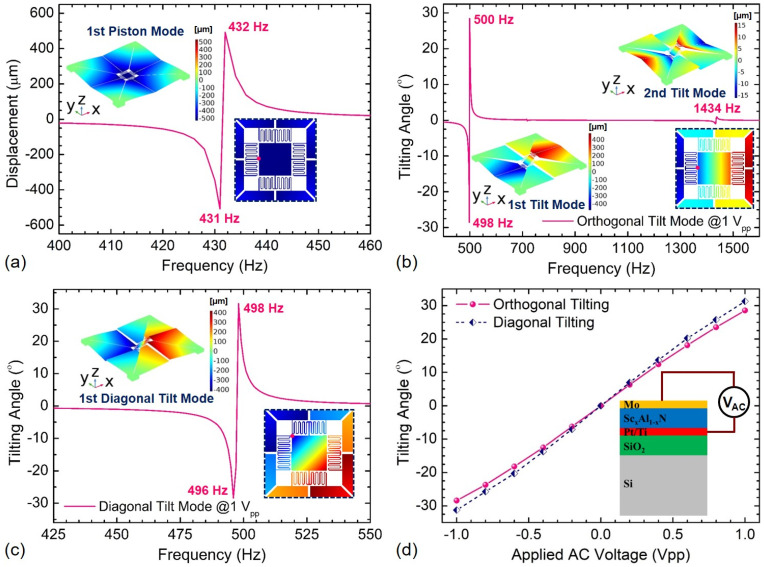
Frequency response of Device A: (**a**) displacement at the piston mode, (**b**) tilting angle at the 1st and 2nd orthogonal tilt modes, (**c**) tilting angle at the diagonal tilt mode, (**d**) tilting angle vs. applied AC voltage.

All PM actuators (X1–X4, Y1–Y4) need be driven by the same alternating-current (AC) voltage if Devices A or B are expected to work at the piston vibration modes. To target the orthogonal 0°-tilt vibration modes (referring to Figure 2), the other PM actuators (X3, X4, Y3, Y4) must be excited by an opposite one when the PM actuators (X1, X2, Y1, Y2) are excited by an AC voltage applied on two electrode surfaces of the Sc_x_Al_1−x_N film. On the other hand, the pair of PM actuators (X1, X4, Y1, Y4) and (X2, X3, Y2, Y3) can be configured with opposite AC voltage excitations to obtain the orthogonal 90°-tilt vibration modes. In addition, Devices A and B can also operate at the diagonal tilt modes when the pair of PM actuators (X1, X2, Y2, Y3) and (X3, X4, Y1, Y4) are excited by the opposite AC voltages. At 1 V_pp_ driving voltage, the simulated displacement responses at the edge of Device A are plotted in Figure 12. This shows that the proposed scanning mirror is able to work very efficiently and powerfully, offering the piston displacement sensitivity of 509 μm/V_pp_ at 431 Hz and the orthogonal tilt displacement sensitivities of 272 μm/V_pp_ at 498 Hz and 8.7 μm/V_pp_ at 1435 Hz. Moreover, the diagonal tilt displacement of 303 μm can be achieved by applying 1 V_pp_ voltage at 498 Hz, in which case the maximum principal stress in Device A is about 426 MPa. Both the orthogonal and diagonal tilting angles increases linearly with the driving voltage, offering the tilting angle sensitivities of 28.6°/V_pp_ and 31.3°/V_pp_ at 498 Hz, respectively, as shown in Figure 12d.

**Figure 13:**
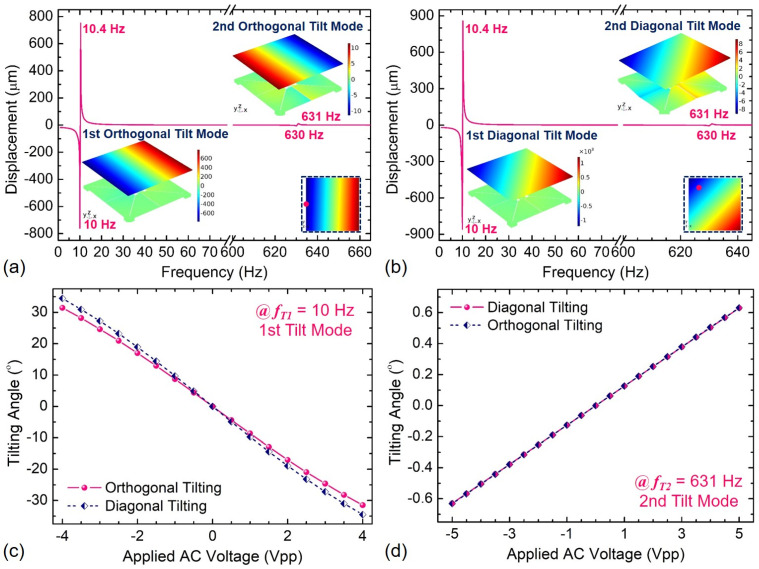
Frequency response of Device B: (**a**,**b**) displacement at the 1st and 2nd tilt modes, (**c**,**d**) tilting angle vs. applied AC voltage.

The dynamic response of Device B was also investigated by simulations, as shown in Figure 13. After reasonable allocation of eight PM actuators, the first-order piston displacement sensitivity is about 1542.2 μm/V_pp_ at 69 Hz, while the first-order orthogonal and diagonal tilting angles reach about 8.1°/V_pp_ and 8.9°/V_pp_ at 10 Hz, respectively. The maximum principal stress at the first-order tilt modes will be as high as 792.6 MPa when the applied AC voltage is 4.0 V_pp_. The resonant frequencies of both the piston and tilt modes decrease dramatically because of the larger micro-mirror mass as compared to that of Device A. The products of *θ·D* for Devices A and B at the first-order tilt mode is about 31.3°·mm/V_pp_ and 88.5°·mm/V_pp_, respectively, which is larger than almost all of those in the literature [4,16,21,43,44,45]. Moreover, the second-order orthogonal and diagonal tilting angles of about 0.126° was obtained when an AC voltage of 1 V_pp_ at 631 Hz was applied, while the maximum principal stress is at level of 160 MPa/V_pp_.

## 5. Conclusions

In this study, a particularly efficient Sc_x_Al_1−x_N-based piezoelectric MEMS scanning mirror with a pupil size of 10 mm was explored. The novel MEMS mirror, comprised a reflection mirror plate, four S-shaped meandering springs and eight trapezoidal Sc_x_Al_1−x_N-based actuators, was proposed to achieve large static and dynamic two-axis tilting angles. The detailed theoretical modeling, simulations and comparative analysis for two different Sc_0.41_Al_0.59_N-based MEMS mirror designs were investigated prior to device fabrication. For the proposed Device A including a square mirror with a 1 mm length and 10 μm thickness, the maximal orthogonal and diagonal static tilting angles were ±36.2°@200 V_DC_ and ±36.2°@180 V_DC_ with the maximum principal stress of less than 767.2 MPa, respectively. In the dynamic actuation regime, the piston displacement sensitivity was 509 μm/V_pp_ at 431 Hz, and both the first-order orthogonal and diagonal tilting angles increased linearly with the driving voltage, offering the tilt angle sensitivities of 28.6°/V_pp_ and 31.3°/V_pp_ at 498 Hz, respectively. In comparison, Device B including a square mirror with a 10 mm length and 100 μm thickness was able to provide the accessible tilting angles of ±36.0°@200 V_DC_ and ±36.9°@180 V_DC_ for horizontal and diagonal actuations, respectively. In the dynamic actuation regime, the first-order orthogonal and diagonal tilting angles of ±8.1°/V_pp_ and ±8.9°/V_pp_ could be obtained at 10 Hz, respectively, while the second-order tilting angles are about ±0.13°/V_pp_ at 631 Hz. Moreover, the displacement sensitivity of Device B at the resonant piston mode was also simulated and discussed. This work has suggested from the view of FEM simulations and mathematical calculations that the right-angle trapezoidal actuators potentially possess excellent mechanical efficiency for possible optoelectronic applications thanks to the novel actuator structure design and the Union Jack-shaped arrangement.

## Figures and Tables

**Table 1:** Comparison between ScAlN, AlN and PbZr_x_Ti_1−x_O_3_ (PZT) piezoelectric materials.

Performance	Sc_0.41_Al_0.59_N [11,27,32]	AlN [11,26]	PZT [11,26]
Material Category	Non-ferroelectric		Ferroelectric
Piezoelectric Coefficient, $e_{31,f}$ [C/m^2^]	~3.16	~1.1	~21
Relative Permittivity, $\varepsilon_{r}$	16.7	10	1300
Figure of Merit (FoM), $e_{31,f}^{2}/\varepsilon_{0}\varepsilon_{r}$ [GPa]	67.5	13.7	38.3
Highest DC Driving Voltage [V]	±200	±200	±30
Directionality	Bidirectional		Unidirectional
CMOS compatibility	Yes		No

**Table 2:** Geometrical parameter specifications of mirror devices A and B.

Parameter	Device A	Device B
Mirror length, *l_A_* or *l_B_* [μm]	1000	10,000
Thickness of the mirror plate, *t_A_* or *t_B_* [μm]	10	100
Length of the pillar, *l_p_* [μm]	*-*	250
Height of the pillar, *h_p_* [μm]	*-*	3500
Length of the PM actuator, *l_1_* [μm]	4025	
Fixed boundary width of the PM actuator, *w_0_* [μm]	700	
Lower width of the PM actuator, *w_1_* [μm]	3035	
Upper width of the PM actuator, *w_2_* [μm]	880	
Length of the meandering spring, *l_m_* [μm]	370	
Width of the meandering spring, *w_m_* [μm]	20	
Spacing pitch of the meandering spring, *w_p_* [μm]	120	
Length of the torsion bar, *l_b_* [μm]	390	
Width of the torsion bar, *w_b_* [μm]	20	
Length of the connecting bar, *l_c_* [μm]	410	
Width of the connecting bar, *w_c_* [μm]	20	

**Table 3:** Material properties used in the simulation of both devices A and B [4,8,16,23,39,40].

Parameter	Si	SiO_2_	Pt	Mo	Au
Young’s Modulus [GPa]	170	70	168	312	70
Poisson’s Ratio	0.28	0.17	0.38	0.31	0.44
Density [kg/m^3^]	2329	2200	21,450	10,200	19,300
Relative Permittivity	11.7	4.2	-	-	-

**Table 4:** Static performance comparison between the MEMS mirrors presented here and in the literature.

Piezoelectric Mirrors	Material	Mirror Size, *D* [mm]	Tilt Angle, *θ* [°/V]	Maximum Angle, *θ_max_* [°]	*θ·D* [°·mm/V]
Device A	ScAlN	1	0.2011	±36.2 @180 V	0.201
Device B	ScAlN	10	0.1999	±36.0 @180 V	1.999
Ref. [11]	ScAlN	0.8	0.0933	±14.00 @150 V	0.075
Ref. [21]	AlN	0.2	0.005	±0.15 @30 V	0.001
Ref. [42]	Bulk PZT	20	0.0224	3.14 @120 V	0.449
Ref. [43]	PZT	2	0.46	4.60 @10 V	0.920
Ref. [44]	PZT	2	0.3	5.10 @17 V	0.600

## Data Availability

Data sharing not applicable.
